# Coverage-level and predictors of maternity continuum of care in Nigeria: implications for maternal, newborn and child health programming

**DOI:** 10.1186/s12884-023-05372-4

**Published:** 2023-01-18

**Authors:** Oyewole Kazeem Oyedele, Adeniyi Francis Fagbamigbe, Odunayo Joshua Akinyemi, Ayo Stephen Adebowale

**Affiliations:** 1 Department of Epidemiology and Medical Statistics, Faculty of Public Health, College of Medicine, University of Ibadan, Ibadan, Nigeria; 2 International Research Centre of Excellence, Institute of Human Virology, Nigeria, Abuja (FCT), Nigeria; 3 Faculty of Humanities, Population Health and Research Entity, North West University, Mafikeng, South Africa

**Keywords:** Maternity continuum of care, Antenatal care, Skilled birth attendants, Postnatal care, Model Pathway, Backward stepwise regression, Complementary-log–log model

## Abstract

**Background:**

Completing maternity continuum of care from pregnancy to postpartum is a core strategy to reduce the burden of maternal and neonatal mortality dominant in sub-Saharan Africa, particularly Nigeria. Thus, we evaluated the level of completion, dropout and predictors of women uptake of optimal antenatal care (ANC) in pregnancy, continuation to use of skilled birth attendants (SBA) at childbirth and postnatal care (PNC) utilization at postpartum in Nigeria.

**Methods:**

A cross-sectional analysis of nationally representative 21,447 pregnancies that resulted to births within five years preceding the 2018 Nigerian Demographic Health Survey. Maternity continuum of care model pathway based on WHO recommendation was the outcome measure while explanatory variables were classified as; socio-demographic, maternal and birth characteristics, pregnancy care quality, economic and autonomous factors. Descriptive statistics describes the factors, backward stepwise regression initially assessed association (*p* < 0.10), multivariable binary logistic regression and complementary-log–log model quantifies association at a 95% confidence interval (α = 0.05).

**Results:**

Coverage decrease from 75.1% (turn-up at ANC) to 56.7% (optimal ANC) and to 37.4% (optimal ANC and SBA) while only 6.5% completed the essential continuum of care. Dropout in the model pathway however increase from 17.5% at ANC to 20.2% at SBA and 30.9% at PNC. Continuation and completion of maternity care are positively drive by women; with at least primary education (AOR = 1.27, 95%CI = 1.01–1.62), average wealth index (AOR = 1.83, 95%CI = 1.48 –2.25), southern geopolitical zone (AOR = 1.61, 95%CI = 1.29–2.01), making health decision alone (AOR = 1.39, 95%CI = 1.16–1.66), having nurse as ANC provider (AOR = 3.53, 95%CI = 2.01–6.17) and taking at least two dose of tetanus toxoid vaccine (AOR = 1.25, 95%CI = 1.06–1.62) while women in rural residence (AOR = 0.78, 95%CI = 0.68–0.90) and initiation of ANC as late as third trimester (AOR = 0.44, 95%CI = 0.34–0.58) negatively influenced continuation and completion.

**Conclusions:**

6.5% coverage in maternity continuum of care completion is very low and far below the WHO recommended level in Nigeria. Women dropout more at postnatal care than at skilled delivery and antenatal. Education, wealth, women health decision power and tetanus toxoid vaccination drives continuation and completion of maternity care. Strategies optimizing these factors in maternity packages will be supreme to strengthen maternal, newborn and child health.

## Introduction

Continuum of Care (CoC) for maternal healthcare involves an integrated system that connects essential maternal, newborn and child health (MNCH) services, throughout preconception, pregnancy, childbirth, postnatal and child care [1]. Strengthening MNCH framework through the integrated CoC model remains an optimum strategic design to accomplish mother and child survival, especially in sub-Saharan Africa (SSA) where the defunct Millennium Development Goals (MDG) 4 and 5 were not achieved by 2015 [2, 3]. The goals are now included in the Sustainable Development Goals (SDG) [4]. CoC that covered antenatal care (ANC), skilled delivery and postnatal care (PNC) services are therefore paramount to attain complication-free pregnancy, optimal health, and as well reduced maternal and neonatal morbidity and mortality [5].

However, most women in SSA including Nigeria either failed to complete the required antenatal, intrapartum and postpartum care or dropout from the CoC [6, 7]. High dropout in combination with other factors explained the high maternal mortality ratio (MMR) in SSA with Nigeria among the top four most affected countries [8]. Whereas, most of the maternal death in SSA that accounted for two-thirds of global MMR are preventable if the WHO recommendations for optimal ANC; through early initiation and a minimum of 4 contacts (and now 8 with specific components like; blood and urine test, tetanus toxoid vaccination, intermittent preventive treatment and so on) in pregnancy, intrapartum care during labor and childbirth and PNC within the first six weeks after births by skilled birth attendants (SBA) for a positive outcome were upheld [9–12].

The current MMR estimate in Nigeria according to WHO is 917 deaths per 100, 000 livebirths [8, 13]. Though the recent population health survey in Nigeria reported MMR as 512 deaths per 100,000 live births and pregnancy-related mortality ratio (PRMR) as 556 deaths per 100,000 livebirths while neonatal mortality rate (NMR) is 39 deaths per 1000 livebirths in 2018 and, thus implying about one death in every 25 livebirths [14]. The slightly lower MMR can be ascribed to a slight increase in ANC coverage from 61% in 2013 to 67% in 2018, skilled delivery increase from 39% in 2013 to 43% in 2018 while PNC coverage stayed at 42% in 2018 [14–16].

Despite the recent increase, Nigeria still fell short of the recommended coverage level for the three major maternity services and such little rise over the years has continued to slow progress in achieving MMR and NMR of less than 70 per 100,000 and 25 per 1000 livebirths by 2030 respectively [4, 17]. MNCH framework that incorporated CoC model strategy was evidently adopted in Egypt (Northern Africa) through programs that double SBA to parturient ratio and improved institutional facilities that encourage ANC and PNC has led to the achievement of more than 90% coverage, up to 50% CoC completion and reduced maternal and neonatal deaths [18, 19].

Parturient in Nigeria are however affected by many factors in the use of maternal health services [17, 20]. Studies have reported that wealth, education, type of residence among others are associated with the underutilization and utilization of ANC in Nigeria [21–23]. Literatures on SBA use in Nigeria highlighted births preparedness, ANC visit, pregnancy complications and women’s involvement in healthcare decisions as major determinants [13, 24–26]. The effect of both ANC and SBA utilization on PNC uptake has been reported [27–29]. Literatures on maternity CoC completion in Nigeria are limited but studies have found that maternity CoC Completion in Ethiopia is associated with ANC initiation within second trimester, secondary education, involving women in healthcare decision and reachable distance to health center [30, 31]. Similar factors in addition to media access, birth order and being informed of signs of pregnancy complications were determinants of continuity of maternity CoC in the Gambia [32]. Whereas, urine sample testing in pregnancy, household wealth status and delivery at a health facility were significantly associated with women’s continuation from use of SBA to PNC after receiving ANC in Cambodia [33].

Though studies have independently investigated ANC, SBA and PNC in Nigeria [21–26, 34], there is however paucity of information on the linkage among the three pillars of maternity CoC. Although Akinyemi et al. assessed dropout, the study did not consider the recommended optimal number of ANC contacts and the pregnancy-related factors [6]. Meanwhile, the policy goal of the healthcare system is to ensure that every pregnant woman receives all essential maternal health services across the pathway of the childbirth. Also, the coverage gap in the completion of maternity CoC based on time dimension (pregnancy to postpartum period) has not been studied in Nigeria.

This study thus adds to the body of knowledge on maternal and child health by considering the optimal ANC contacts recommended by WHO, delivery assisted by SBA, and first PNC within the first 48 h after childbirth in investigating the levels of coverage of maternity continuum of care and its determinants in Nigeria. In this study, we answered the following questions; What is the level of coverage of the maternity continuum of care in Nigeria? Is the rate of dropout from maternity healthcare similar along the continuum of care pathway? What are the socio-demographics and maternal health characteristics associated with the maternity continuum of care in Nigeria? The research findings will provide evidence-based information for MNCH programs that will support policy decisions toward strengthening pregnancy, childbirth, and puerperium care in Nigeria.

## Methodology

### Study design, data and area

**Fig. 1:**
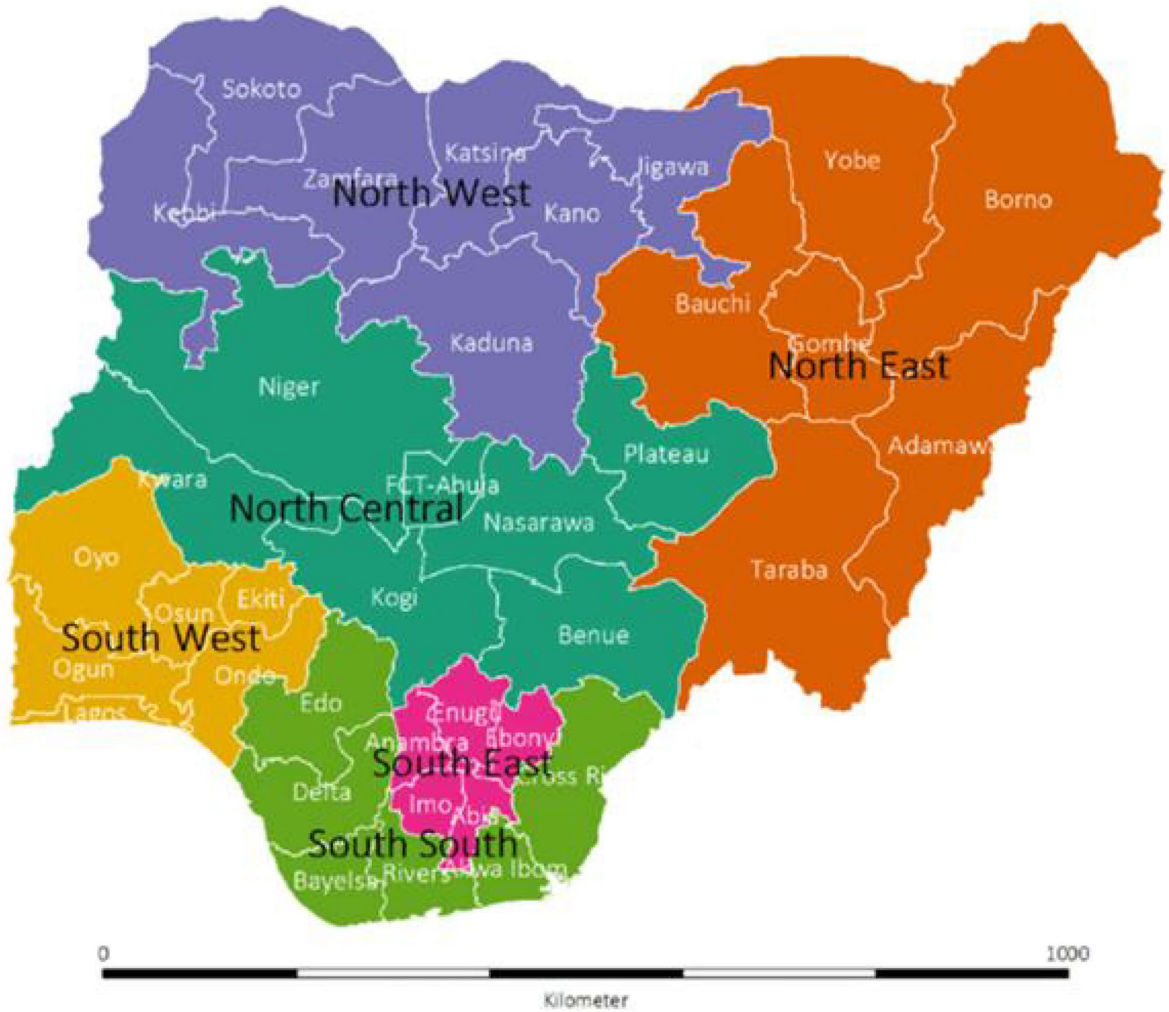
Map of Nigeria showing the 36 states and FCT by the geopolitical zones

The study is a secondary analysis of 2018 Nigerian Demographic and Health Survey (NDHS) data. NDHS is a cross-sectional population-based and nationally representative survey routinely collected in five years’ intervals in Nigeria. Nigeria is administratively grouped into six geopolitical zones (Northcentral, Northeast, Northwest, Southeast, Southsouth and Southwest) with an average of 6 states per geo-political zone and the federal capital territory (FCT) as the administrative headquarter [14]. Each state is further divided into local government areas that serve as the lowest and the closest administrative cadre of government for the people. The 36 states and FCT are shown in the study area map in Fig. 1.

### Sampling strategy and participants

The sampling frame of the 2018 nationally representative NDHS was obtained from the list of rural and urban enumeration areas collated by the National Population and Housing Census (NPHC) in Nigeria. A two-stage stratified random sampling design was used in the 2018 NDHS, where 1400 enumeration areas consisting of 820 rural and 580 urban strata were selected using probability proportional to size at the first sampling stage. Hence the difference in the number of urban and rural strata. Equal probability systematic sampling was then used to select the same number of households (30 households per enumeration area) in the second sampling stage. A total of 41,821 (22,658 in rural and 19,163 in urban) women participants were interviewed in the cross-sectional survey that achieve a 99% response rate [14]. 21,447 women who had at least one ANC visit and whose information were at least non-missing in one of the maternity CoC pathway made up the weighted sample size of the study. The survey also collected information on women’s demographics, socioeconomic and health-related characteristics that includes the key measures of the maternity continuum of care (ANC, SBA and PNC) investigated in this study.

### Outcome variables

Outcomes of interest in this study are the maternity continuum of care received during pregnancy (ANC), childbirth (use of SBA) and post-delivery (PNC). A postpartum woman is regarded to have completed the three gamut of care if she received the recommended 4 or more ANC contacts in a healthcare facility during pregnancy, move on to utilize SBA i.e., delivery assisted by at least a doctor, nurse or midwive and subsequently received postnatal checkup within the first 48 h after childbirth [14]. The combined outcome was based on the WHO recommendation of at least 4 ANC visits and the use of SBA at birth, especially in low-resource settings of the lower-middle-income countries [11, 12]. We measured PNC within the first two days after birth which has been reported in the 2018 NDHS due to most maternal morbidity and mortality that occur at the time and therefore highlighted PNC (within two days) as an important measure in the maternity CoC model [33]. We avoided the adaptation of the recently recommended 8 ANC contacts since the DHS framework was designed on a minimum of 4 ANC visits as the optimal number of ANC visit and also; because the strategy to implement the 8 ANC visits was recently devised in the orientation package for healthcare providers in Nigeria after most of the respondents have had the indexed childbirth [14, 35, 36]. The outcome variable was obtained from the combination of responses to the following questions:

- 1. How many times did you receive antenatal care during this pregnancy?
- 2. Who assisted with the delivery of (NAME)?
- 3. Did anyone check on your health after you left the facility i.e., the place of delivery?

Three sets of dichotomous variables were extracted, such that; a positive response to question ‘1’ is 4 or more ANC and negative response is ANC visit less than 4 (0, 1, 2, 3), response to question ‘2’ that delivery was assisted by doctor/nurse/midwife is a positive response and otherwise a negative response and similarly positive response to question ‘3’ is ‘Yes’ and ‘No’ is the negative response. The sequence of maternity continuum of care was drawn from the combination of positive responses. Hence, positive response to; question 1 indicate ANC (4 +) visits, question 2 indicate ANC (4 +) visits and SBA use and question 3 indicate maternity CoC completion in this study i.e., when ANC (4 +), SBA and PNC were all received.

### Explanatory variables

Independent variables included in this study were based on similar factors considered by previous studies that investigated the maternity continuum of care [3, 5, 30–33, 37]. This can be defined under the broad categories as; socio-demographic characteristics, maternal health and birth factors, quality of pregnancy care received, economic status and physical and autonomy factors [13, 38, 39].

#### Socio-demographic characteristics

These includes maternal age (15–24, 25–34, 35–49 years), place of residence (urban, rural), educational level (none, primary, secondary, tertiary), marital status (never married, married, cohabiting, divorced/widowed/separated) husband educational level (none, primary, secondary, tertiary), geopolitical zone (northcentral, northeast, northwest, southeast, south-south, southwest).

#### Maternal health and birth factors

These are birth-related and women health-seeking characteristics. Which are; wanted last pregnancy (wanted then, wanted later, wanted no more), birth order (1, 2, 3 and 4 +), covered by health insurance (no, yes), the timing of first ANC visit (first, second and third trimester), institutional delivery (yes, no), delivery by caesarian section mode (yes, no), childbirth sex (male, female), child-size at birth (very small, smaller than average, average, larger than average, very large).

#### Quality of pregnancy care received

These are factors assessing pregnancy care which are; status of blood pressure measured during pregnancy (yes, no), urine sample taken during pregnancy (yes, no), blood sample taken during pregnancy (yes, no), iron-folic acid tablet taken during pregnancy (yes, no), number of tetanus toxoid vaccine taken during pregnancy (0, 1, 2 +), provider of ANC (no one/traditional birth attendant, community health ‘extension’ worker, auxiliary nurse/midwife, skilled nurse/midwife, doctor).

#### Economic status

Employment type (not-working/manual/clerical, agricultural, sales, services, professional/ managerial/technical/), Wealth index (poor, average, rich), Media access (no, yes).

#### Healthcare accessibility and autonomy factors

Distance to health facility (no problem, big problem), Person who usually decides on respondent’s healthcare (respondent alone, both, spouse alone), Person who usually decides on how respondent’s earnings are spent (partner alone, joint decision, respondent alone).

### Statistical analysis

Descriptive statistics of the background characteristics and outcomes were reported in frequency and percentages. Missing data were reported for at least 1% of the observation and otherwise negligible i.e., less than 1%. Three sequences of maternity CoC model defined under the space that; postpartum women received at least 4 ANC visits during pregnancy was coded as 1 and 0 otherwise – model 1, continued from ANC (4 +) to use SBA at childbirth was coded as 1 and 0 otherwise – model 2 and completed the three key CoC which is from ANC (4 +) to SBA and to PNC after childbirth was equally coded as 1 and 0 otherwise – model 3 were fitted.

Initially, model selection was carried out to assess the set of maternal factors/characteristics associated with the maternity CoC model (models 1, 2 and 3). This was carried out using the backward stepwise logistic regression for models 1 and 2 and backward stepwise complementary log–log regression for model 3 due to the rare outcome and since the probability of completing the three key maternity continuum of care is small (less than 10%). The backward regression started with the full model and at each model step, the variable whose removal significantly reduced the log likelihood (-2logL) was returned and retained in the model and otherwise removed. All the independent variables were given an equal chance of selection and variable inclusion was considered at *p *< 0.10.

Bivariate and Multivariate analysis that includes all the significant variables retained in the stepwise regression (final models 1, 2 and 3) were performed to determine the likelihood and significance of each of the predictor variables and the combined set of the predictors respectively. The respective unadjusted and adjusted odds ratio were reported for the binary logistic regression analysis of models 1 and 2 while the unadjusted and adjusted e(form) or exp(b) equivalent of the odds ratio was reported in the multivariable complementary log–log analysis of model 3. Data were weighted with the women’s sample weight indices included in the NDHS data and the svyset command was used to adjust for unequal group/population sizes due to the complex survey design. Bivariable and multivariable statistical analysis were performed at 10% and 5% level of significance (95% confidence level) respectively, using Stata version 16.0 (Stata Corp, Texas, USA). Variable (Union type) that causes multicollinearity (variance inflation factor > 5) was subsequently removed from the multivariate analysis.

### The multivariable regression analysis

The multiple binary logistic regression and the complementary log–log modeled the odds of optimal ANC uptake and continuation to the use of SBA and PNC as a binary response [P($${Y}_{i}=0$$), P($${Y}_{i}=1)]$$ [40, 41]. The multiple logistic model which equates the function of the odds to a linear combination of the regression terms and the predictors is generally expressed as:

1 $${Y}_{i}= \mathrm{ln}\left(\frac{P}{1-P}\right)= {\beta }_{0}+ {\beta }_{1}{X}_{1i}+\dots + {\beta }_{p}{X}_{pi}+ \varepsilon$$

2 $$E\left({Y}_{i}\right)={P}_{i}=\frac{\mathrm{exp}\left({\beta }_{0}+{\beta }_{1}{x}_{1i}+\cdots +{\beta }_{p}{x}_{pi}\right)}{1+\mathrm{exp}\left({\beta }_{0}+{\beta }_{1}{x}_{1i}+\cdots +{\beta }_{p}{x}_{pi}\right)}$$

where: $\mathrm{ln}\left(\frac{P}{1-P}\right)$ is the log odds (P is the probability of success and 1-P is the failure probability).

${\beta }_{0}$ is the logistic regression constant.

${\beta }_{1}+\dots + {\beta }_{p}$ are the px1 vector of regression coefficient or estimates of the multiple predictors.

${X}_{i1}+\dots +{X}_{ip}$ are the nxp matrix of explanatory variables predicting the log odds in the model.

When the probability of success “P” is very large or very small (less than 10%) leading to asymmetrical S-shape compared to the symmetric logistic curve [42], the use of the complementary log–log model becomes more appropriate (accurate) as it’s in rare CoC outcome. The complementary-log–log model is generally stated as:

3 $${Y}_{i}=\mathrm{log}\left\{-\mathrm{log}\right.\left.\left[1-\pi \left(\mathrm{x}\right)\right]\right\}={\beta }_{0}+{\beta }_{1}{X}_{1i}+\cdots +{\beta }_{p}{X}_{pi}+\varepsilon$$

4 $$\mathrm E\left(Y_i\right)=\pi\left(\mathrm x\right)=1-\exp\left[-\exp\left(\beta_1X_{1i}+\cdots+\beta_pX_{pi}\right)\right]$$

where log{-log[1-π(x)]} is the complementary log–log transformation with binary response (0, 1).

## Results

### Maternity continuum of care model pathway

**Fig. 2:**
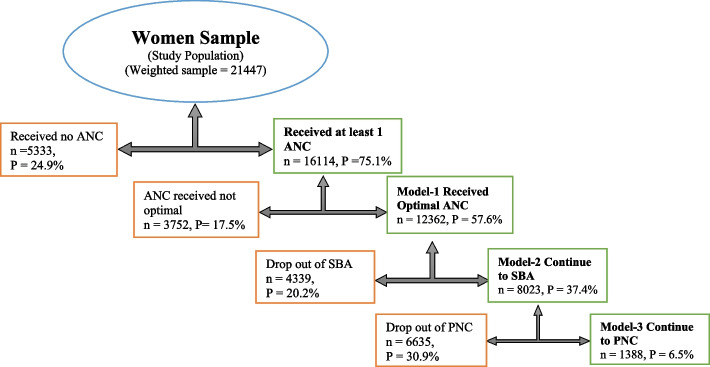
Model pathway showing continuation to and dropout from maternity continuum of care in Nigeria

Figure 2 shows the pathway of the continuum of care model (from pregnancy to delivery and to postpartum), based on the key maternity health service received at each stage. Antenatal care assessed maternal health service received in pregnancy, skilled birth attendant utilization at delivery and postnatal care at postpartum. Among the 21,447 pregnancies reported in NDHS 2018, 75.1% (16,114) received antenatal care at least once. Antenatal care was optimal in this study when a postpartum woman received at least 4 contacts (*n* = 12,362, *P* = 57.6%) which made up pregnant women’s inclusion in the first stage of the CoC model (Model-1).

About 37.4% (8023) of Pregnant women continued to use skilled birth attendants at childbirth. Implying that 20.2% (4339) who had delivery performed by unskilled births attendants after receiving optimal antenatal care drop out at the second stage of the CoC model. Continuation to use of postnatal care service at the third stage after receiving optimal ANC and SBA indicates completion of the CoC by only 6.5% (1388) while 30.9% (6635) dropped out from postnatal care service after receiving optimal ANC and SBA. Dropout from any of the CoC models at any stage along the pathway will lead to incomplete receipt of the essential maternal health service across pregnancy to the puerperium continuum. The CoC model pathway is shown in Fig. 2.

### Background characteristics

Table 1 shows the percentage distribution of women who had at least one birth in the last 5 years preceding the survey by background characteristics. About 25% of the women respondents were in the early maternal age (15–24 years), while 27.5% were in the late maternal age (35–49 years). Nine of ten (91.4%) were married, 2.8% were cohabiting while 3.5% are either divorced or widowed. 45.2% and 34.1% of women and their partner has no education while only 8.5% and 14.2% of women and their partners have completed higher education respectively. 61.0% of respondents resides in the urban area and, are not exposed to mass media and only 28.1% belonged to the rich wealth quintile. Most (35.6%) of the women respondents were from the northwest geopolitical zone compared to the few (9.0%) from the south-south (Table 1).

**Table 1:** Descriptive analysis of women characteristics

Maternal Characteristics	Frequency Weighted Sample (*n* = 21,447)	Percentage (%)
**Maternal Age**		
15 – 24	5321	24.8
25 – 34	10,232	47.7
35 – 49	5894	27.5
**Level of education**		
None	9703	45.2
Primary	3211	15.0
Secondary	6699	31.2
Higher	1834	8.5
**Marital Status**		
Never married	485	2.3
Married	19,610	91.4
Cohabiting	613	2.8
Divorced/Separated/Widowed	739	3.5
**Partner’s level of education**		
No education	7317	34.1
Primary	2787	12.9
Secondary	6773	31.6
Higher	3036	14.2
Missing	1534	7.2
**Place of residence**		
Urban	8372	39.0
Rural	13,076	61.0
**Employment type**		
Not working/Manual/Clerical	984	4.6
Agricultural	3226	15.1
Sales	8687	40.5
Services	1394	6.5
Professional/Managerial/Technical/Service	1118	5.2
Missing	6036	28.1
**Wealth status**		
Poor	9521	44.4
Middle	4378	20.4
Rich	7548	35.2
**Media exposure**		
No	13,017	60.7
Yes	8430	39.3
**Geopolitical zone**		
North Central	3008	14.0
North East	3845	17.9
North West	7633	35.6
South East	2053	9.6
South south	1925	9.0
South West	2983	13.9
**Wanted pregnancy**		
Then	18,881	88.0
Later	1855	8.7
No more	711	3.3
**Birth order**		
1	3646	17.0
2	3842	17.9
3	3250	15.2
4 +	10,709	49.9
**Covered by health insurance**		
No	20,978	97.8
Yes	469	2.2
**Getting permission to go for medical help**		
Not a big problem	18,886	88.1
Big problem	2561	11.9
**Getting money for medical help**		
Not a big problem	11,051	51.5
Big problem	10,396	48.5
**Getting medical help; distance to facility**		
Not a big problem	15,406	71.8
Big problem	6041	28.2
**Woman healthcare decision maker**		
Partner alone	12,052	56.2
Woman alone	1895	8.8
Joint decision	6222	29.0
Missing	1278	6.0
**Provider of ANC**		
TBA/No-one	5666	26.4
CHEW/CHW	1682	7.8
Auxiliary Nurse/Midwife	508	2.4
Nurse/Midwife	12,186	56.8
Doctor	1405	6.6
**Timing of first ANC**		
1^st^ trimester	4008	17.9
2^nd^ trimester	10,411	47.4
3^rd^ trimester	2123	9.8
Missing	5350	24.9
**BP measured during ANC**		
No	966	4.5
Yes	15,148	70.6
Missing	5333	24.9
**Blood sample taken during ANC**		
No	2005	9.3
Yes	14,109	65.8
Missing	5333	24.9
**Urine sample taken during ANC**		
No	2215	10.3
Yes	13,899	64.8
Missing	5333	24.9
**Iron folic acid taken during ANC**		
No	6612	30.8
Yes	14,793	69.0
**Tetanus toxoid vaccine taken in ANC**		
0	6501	30.0
1	3600	16.9
2 +	11,261	53.1
**Institution delivery**		
No	12,784	59.6
Yes	8664	40.4
**Delivery by CS**		
No	20,778	96.9
Yes	669	3.1
**Child sex at birth**		
Male	10,967	51.2
Female	10,480	48.8
**Child size at birth**		
Very small	593	2.8
Smaller than average	2337	10.9
Average	10,901	50.8
Larger than average	5408	25.2
Very large	1899	8.9
Missing	309	1.4
**Total**	**21,447**	**100.0**

*TBA* Traditional Birth Attendants, *CHEW* Community Health Extension Worker, *CHW* Community Health Worker

About 88% of the women wanted the pregnancy and about 50% have had at least four births. Only 2.2% were covered by health insurance while about 48.5 and 28.2% reported big problems in getting money for medical help and in reaching medical facilities respectively (Table 1). Most (56.2%) of women’s healthcare decisions were made by the partner while only 8.8% of the women made their healthcare decision alone. Nurse/Midwives were the providers of ANC for 56.8% of the women while 26.4% either had no-one or utilized TBA (Table 1). Only nearly 18% had first ANC in 1^st^ trimester. 70.6, 65.8 and 64.8% of women had their blood pressure measured, blood and urine sample taken at ANC respectively. 69 and 53.1% of the women took iron folic acid and at least 2 dose of tetanus vaccine during pregnancy respectively. 40.4% of the women delivered at a hospital and 3.1% of them were through Caesarian mode. 51.2% of the women recently delivered a male child while 48.8% delivered a female child. About 2.8% of the women delivered a child that is very small in size while 8.9% delivered a very large child (Table 1).

### Retention in the maternity continuum of care

**Fig. 3:**
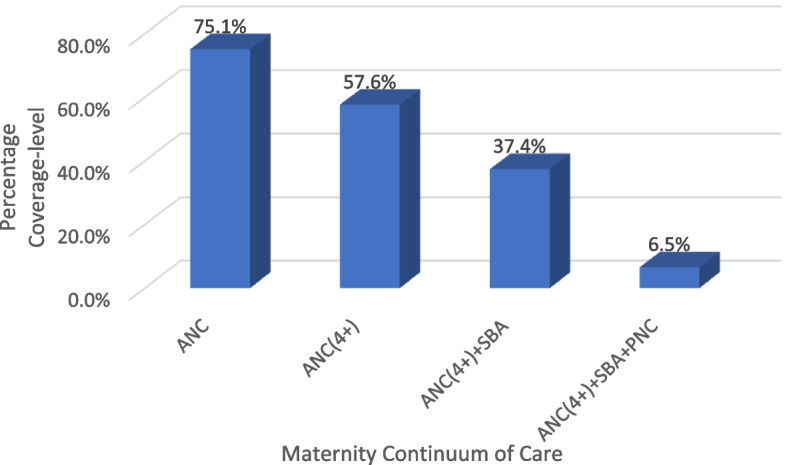
Coverage-level of maternity continuum of care by women with at least a birth

Figure 3 shows that only 6.5% of the women population completed the maternity continuum of care (received optimal ANC, continue to use SBA at childbirth and received PNC service after delivery) while 93.5% of the women had an incomplete maternity continuum of care (at least one of the optimal ANC, SBA and PNC service was not received). 37.4% received optimal ANC and SBA, 57.6% received Optimal ANC only and 75.1% received at least one ANC (Fig. 3).

### Modelling factors associated with the continuum of care

The result of backward stepwise regression in Table 2 highlighted selected variables associated with women uptake of optimal ANC (4 +) (model 1), continuation to use of SBA (model 2) and the use of PNC (model 3). In model 1, marital status (-2logL = 2.049, *p* = 0.152) and Employment type (-2logL = 0.855, *p* = 0.355), and other variables whose inclusion led to insignificant change in -2logL were removed from the full model 1 while the remaining variables whose removal led to significant (*p* < 0.10) change in -2logL were retained (Table 2). Similarly, institutional delivery (-2logL = 2341.697, *p* = 0.000) along with other variables led to a significant (*p* < 0.10) change in -2logL and were therefore retained in model 2. Also, the removal of women and partner education, women healthcare decider, provider of ANC, time of first ANC and others that led to a significant change in -2logL (Table 2) were subsequently retained in the final model 3 (CoC completion).

**Table 2:** Backward stepwise model selection of factors associated with maternity continuum of care

Maternal Factors	Model-1 ANC(4 +)			Model-2 ANC(4 +) + SBA			Model-3 ANC(4 +) + SBA + PNC		
	B	Change in -2logL	*p*-value	B	Change in -2logL	*p*-value	B	Change in -2logL	*p*-value
Maternal Age	0.219	19.425^a^	0.000	0.289	26.148^a^	0.000	0.053	0.769	0.381
Education	0.192	24.992^a^	0.000	0.365	93.257^a^	0.000	0.209	20.465^a^	0.000
Marital Status	-0.244	2.049	0.152	-0.268	2.743^a^	0.098	0.265	3.433^a^	0.060
Partner education	0.076	5.196^a^	0.023	0.036	0.840	0.359	0.144	11.203^a^	0.001
Residence	-0.114	2.832^a^	0.092	-0.230	10.341^a^	0.001	-0.312	16.449^a^	0.000
Employment	0.031	0.855	0.355	0.009	0.061	0.804	-0.028	0.763	0.382
Wealth status	0.250	32.595^a^	0.000	0.346	58.017^a^	0.000	0.235	16.933^a^	0.000
Media exposure	0.004	0.004	0.950	0.092	1.879	0.170	0.116	2.477	0.116
Geopolitical zone	0.320	231.992^a^	0.000	0.366	292.878^a^	0.000	0.098	24.408^a^	0.000
Wanted pregnancy	-0.035	0.298	0.584	0.016	0.061	0.805	0.107	2.504	0.103
Birth order	-0.092	7.759^a^	0.005	-0.159	19.777^a^	0.000	-0.045	2.124	0.145
Health insurance	0.035	0.024	0.878	-0.170	0.560	0.454	0.009	0.003	0.957
Getting Permission for medical help	-0.138	1.806	0.179	-0.008	0.004	0.949	-0.006	0.002	0.966
Getting money for medical help	-0.115	4.114^a^	0.043	-0.104	2.225	0.136	-0.109	3.104^a^	0.096
Medical help distance to facility	-0.054	0.562	0.453	0.126	2.745^a^	0.098	0.148	2.388	0.122
Woman healthcare decision maker	0.100	9.794^a^	0.002	0.122	12.284^a^	0.000	0.081	4.899^a^	0.022
Provider of ANC	0.159	22.789^a^	0.000	0.699	289.904^a^	0.000	0.399	59.237^a^	0.000
Timing of 1^st^ ANC	-1.746	997.383^a^	0.000	-1.034	327.770^a^	0.000	-0.374	46.333^a^	0.000
BP checked in ANC	0.146	1.395	0.238	-0.061	0.147	0.702	-0.289	3.322^a^	0.080
Blood sample taken in ANC	-0.161	2.674	0.102	-0.065	0.426	0.514	0.138	0.927	0.336
Urine sample taken in ANC	0.303	15.794^a^	0.000	0.044	0.140	0.709	0.057	0.145	0.703
Iron folic acid taken in ANC	-0.061	0.384	0.537	-0.159	2.320	0.128	0.702	32.250^a^	0.000
Tetanus toxoid vaccine taken	0.450	128.494^a^	0.000	0.280	32.562^a^	0.000	0.156	6.885^a^	0.006
Institution delivery				3.144	2341.697^a^	0.000	-0.589	57.662^a^	0.000
Delivery by CS				0.071	0.131	0.718	0.409	16.404^a^	0.000
Child sex at birth							0.059	0.815	0.367
Child size at birth							-0.024	0.396	0.529

^a^Significant at *p* < 0.10

### Multivariable regression analysis

#### Predictors of receiving optimal ANC (4 +) contacts among study participants

Model 1 (presented in Tables 3 and 4) shows the regression analysis of the predictors of optimal ANC (4 +) received by women who had at least one birth in the last five years preceding the survey. Average (UOR = 2.72, 95%CI = 2.52–2.93; AOR = 1.21, 95%CI = 1.06–1.37) and rich (UOR = 7.69, 95%CI = 7.16–8.27; AOR = 1.30, 95%CI = 1.11–1.51) women are more likely to receive 4 ANC than poor women. The odds of receiving at least 4 ANC increase by women and partner education and decrease by age and birth order. The place of residents and region were other socio-demographics predictors of receiving at least 4 ANC services (Tables 3 and 4).

**Table 3:** Unadjusted odds ratio of the association between maternity continuum of care by women characteristics

Maternal Factors	Model-1 ANC(4 +)		Model-2 ANC(4 +) + SBA		Model-3 ANC(4 +) + SBA + PNC	
	UOR	95%CI	UOR	95%CI	UOR	95%CI
**Maternal Age**						
15 – 24#	1		1			
25 – 34	1.43***	1.33–1.52	1.67***	1.56–1.79		
35 – 49	1.30***	1.20–1.40	1.64***	1.51–1.77		
**Level of education**						
None#	1		1		1	
Primary	3.36***	3.09–3.66	5.34***	4.84–5.88	4.09***	3.33–5.04
Secondary	6.90***	6.42–7.42	14.7***	13.5–15.9	6.73***	5.66–7.99
Higher	22.4***	18.8–26.7	58.3***	50.1–67.8	11.4**	9.39–13.8
**Marital Status**						
Never married#			1		1	
Married			0.61	0.51–1.37	0.74	0.54–1.02
Cohabiting			1.26	0.99–1.60	1.28	0.86–1.91
Divorced/Separated/Widowed			0.93	0.74–1.17	0.76	0.49–1.16
**Partner’s level of education**						
No education#	1				1	
Primary	3.43***	3.13–3.76			5.25***	4.06–6.81
Secondary	6.22 ***	5.77–6.69			8.58***	6.86–10.7
Higher	10.5***	9.4–11.6			11.1***	8.83–14.1
**Place of residence**						
Urban#	1		1		1	
Rural	0.26***	0.25–0.29	0.19***	0.18–0.20	0.31***	0.27–0.35
**Wealth status**						
Poor#	1		1		1	
Middle	2.72***	2.52–2.93	4.11***	3.77–4.48	3.62***	3.01–4.35
Rich	7.69***	7.16–8.27	15.3***	14.1–16.5	6.67***	5.69–7.82
**Geopolitical zone**						
North Central#	1		1		1	
North East	0.65***	0.59–0.72	0.33***	0.30–0.37	0.62***	0.49–0.78
North West	0.61***	0.56–0.66	0.24***	0.22–0.27	0.49***	0.40–0.91
South East	4.42***	3.85–5.08	4.50***	3.97–5.09	2.73***	2.26–3.32
South south	2.19***	1.94–2.49	1.96***	1.74–2.20	1.78***	1.43–2.19
South West	7.35***	6.39–8.44	6.08***	5.41–6.83	2.73***	2.27–3.27
**Birth order**						
1#	1		1			
2	0.96	0.87–1.06	0.88**	0.80–0.96		
3	0.89*	0.80–0.98	0.81***	0.73–0.89		
4 +	0.54***	0.50–0.59	0.42***	0.38–0.45		
**Getting money for medical help**						
Not a big problem#	1				1	
Big problem	0.54***	0.51–0.57			0.62***	0.55–0.69
**Distance to medical facility**						
Not a big problem#			1			
Big problem			0.52***	0.48–0.5		
**Woman healthcare decision maker**						
Partner alone#	1		1		1	
Woman alone	3.01***	2.70–3.35	3.25***	2.94–3.59	2.87***	2.43–3.39
Joint decision	3.35***	3.13–3.59	4.38***	4.10–4.68	2.72***	2.41–3.07
**Provider of ANC**						
TBA/No-one#	1		1		1	
CHEW/CHW	24.5***	21.0–28.6	7.5	5.58–10.1	5.38***	2.80–10.4
Auxiliary Nurse/Midwife	44.4***	35.4–55.5	63.6***	47.2–85.5	53.3***	30.2–93.9
Nurse/Midwife	68.4***	60.2–77.7	94.6***	74.3–120.5	34.2***	20.4–57.3
Doctor	121.2***	99.8–147.2	262.7***	200.8–343.7	76.5***	45.1–129.5
**Timing of first ANC**						
1^st^ trimester#	1		1		1	
2^nd^ trimester	0.27***	0.23–0.31	0.43***	0.40–0.47	0.58***	0.52–0.66
3^rd^ trimester	0.03***	0.02–0.03	0.08***	0.07–0.09	0.25***	0.19–0.33
**Blood Pressure checked in ANC**						
No#					1	
Yes					1.54**	1.17–2.01
**Urine sample taken in ANC**						
No#	1					
Yes	2.42***	2.19–2.66				
**Iron folic acid taken in ANC**						
No#					1	
Yes					7.02***	5.64–8.73
**Tetanus toxoid vaccine taken in ANC**						
0#	1		1		1	
1	11.3***	10.2–12.5	9.89***	8.73–11.2	5.93***	4.55–7.75
2 +	32.1***	29.3–35.0	20.4***	18.2–22.8	9.17***	7.21–11.7
**Institution delivery**						
No#			1		1	
Yes			52.4***	48.2–57.1	2.56***	2.29–2.85
**Delivery by CS**						
No#					1	
Yes					3.30***	2.75–3.97

^***^ *p* < 0.001

^**^ *p* < 0.01

^*^ *p* < 0.05

^#^ Reference category

*TBA* Traditional Birth Attendants, *CHEW* Community Health Extension Worker, *CHW* Community Health Worker

**Table 4:** Adjusted odds ratio of the association between maternity continuum of care by women characteristics

Maternal Factors	Model-1 ANC(4 +)		Model-2 ANC(4 +) + SBA		Model-3 ANC(4 +) + SBA + PNC	
	AOR	95%CI	AOR	95%CI	AOR	95%CI
**Maternal Age**						
15 – 24#	1		1			
25 – 34	1.19*	1.02–1.37	1.21*	1.02–1.42		
35 – 49	1.41***	1.18–1.69	1.59***	1.29–1.95		
**Level of education**						
None#	1		1		1	1
Primary	1.04	0.89–1.19	1.34**	1.13–1.58	1.27*	1.01–1.62
Secondary	1.22**	1.05–1.42	1.91**	1.63–2.23	1.49**	1.18–1.86
Higher	1.78***	1.36–2.32	2.76***	2.16–3.52	1.81***	1.38–2.37
**Marital Status**						
Never married#			1		1	1
Married			––––	––––	––––-	–––-
Cohabiting			0.75	0.55–1.02	1.25	0.97–1.64
Divorced/Separated/Widowed			––––	––––	––––-	–––-
**Partner’s level of education**						
No education#	1				1	1
Primary	1.13	0.98–1.32			1.84***	1.38–2.43
Secondary	1.23**	1.07–1.41			2.03***	1.56–2.63
Higher	1.20*	1.01–1.44			2.05***	1.54–2.72
**Place of residence**						
Urban#	1		1		1	1
Rural	0.86*	0.76–0.97	0.75**	0.65–0.85	0.78**	0.68–0.90
**Wealth status**						
Poor#	1		1		1	1
Middle	1.21**	1.06–1.37	1.45***	1.24–1.68	1.83***	1.48–2.25
Rich	1.30**	1.11–1.51	1.73***	1.43–2.05	1.75***	1.41–2.17
**Geopolitical zone**						
North Central#	1		1		1	1
North East	0.83*	0.71–0.97	0.68**	0.54–0.85	1.06	0.82–1.37
North West	1.29**	1.11–1.50	0.92	0.74–1.14	0.88	0.70–1.11
South East	1.83***	1.47–2.27	1.74***	1.34–2.24	1.61***	1.29–2.01
South south	2.78***	2.17–3.58	3.13***	2.37–4.14	1.25	0.99–1.59
South West	5.75***	4.51–7.32	5.04***	3.84–6.61	1.68***	1.37–2.06
**Birth order**						
1#	1		1			
2	0.91	0.77–1.08	0.73**	0.58–0.92		
3	0.81*	0.67–0.97	0.53**	0.53–0.86		
4 +	0.78*	0.65–0.94	0.63***	0.50–0.79		
**Getting money for medical help**						
Not a big problem#	1				1	1
Big problem	0.89*	0.81–0.99			0.91	0.81–1.02
**Distance to medical facility**						
Not a big problem#			1	1		
Big problem			1.03	0.88–1.22		
**Woman healthcare decision maker**						
Partner alone#	1		1	1	1	
Woman alone	1.30**	1.08–1.54	1.11	0.78–1.57	1.39***	1.16–1.66
Joint decision	1.20**	1.06–1.34	1.26**	1.06–1.50	1.19**	1.05–1.37
**Provider of ANC**						
TBA/No-one#	1		1		1	
CHEW/CHW	0.69	0.45–1.08	0.95	0.49–1.86	0.88	0.43–1.81
Auxiliary Nurse/Midwife	0.98	0.61–1.59	12.5***	6.03–26.1	5.95***	3.23–10.9
Nurse/Midwife	1.14	0.75–1.74	9.50***	5.12–17.6	3.53***	2.01–6.17
Doctor	0.94	0.59–1.48	7.54***	4.10–13.8	5.60***	3.15–9.93
**Timing of first ANC**						
1^st^ trimester#	1		1		1	
2^nd^ trimester	0.32***	0.27–0.37	0.52***	0.44–0.62	0.74***	0.66–0.84
3^rd^ trimester	0.04***	0.03–0.05	0.08***	0.06–0.10	0.44***	0.34–0.58
**Blood Pressure checked in ANC**						
No#					1	
Yes					0.81	0.60–1.10
**Urine sample taken in ANC**						
No#	1					
Yes	1.54***	1.34–1.75				
**Iron folic acid taken in ANC**						
No#					1	
Yes					1.98***	1.57–2.49
**Tetanus toxoid vaccine taken in ANC**						
0#	1		1	1	1	
1	0.93	0.79–1.10	0.92	0.69–1.22	1.03	0.78–1.37
2 +	2.03***	1.75–2.36	1.52**	1.19–1.94	1.25*	1.06–1.62
**Institution delivery**						
No#			1	1	1	
Yes			26.3***	22.1–31.2	0.54***	0.47–0.62
**Delivery by CS**						
No#					1	
Yes					1.57***	1.28–1.91

^***^ *p* < 0.001

^**^ *p* < 0.01

^*^ *p* < 0.05

^#^ Reference category

*TBA* Traditional Birth Attendants, *CHEW* Community Health Extension Worker, *CHW* Community Health Worker

Healthcare decisions made alone by the women increase the odds of receiving at least 4 ANC by 30% compared to healthcare decisions made by the partner alone (AOR = 1.30, 95%CI = 1.08–1.54) (Table 4). Getting money for medical help however decrease the odds when it’s a big problem (UOR = 0.54, 95%CI = 0.51–0.57; AOR = 0.89, 95%CI = 0.81–0.99). ANC provided by the doctor was strongly associated with at least 4 ANC uptake when other variables were unadjusted (UOR = 121.2, 95%CI = 99.8–147.2) (Table 3). Women are 3 and 25 times less likely to receive at least 4 ANC when the first ANC received was in the second (UOR = 0.27, 95%CI = 0.23–0.31; AOR = 0.32, 95%CI = 0.27–0.37) and third (UOR = 0.03, 95%CI = 0.02–0.03; AOR = 0.04, 95%CI = 0.03–0.05) trimester respectively (Tables 3 and 4). Urine sample (AOR = 1.54, 95%CI = 1.34–1.75) and not less than 2 tetanus toxoid vaccine doses (AOR = 2.03, 95%CI = 1.75–2.36) taken in ANC approximately twice increase the odds of receiving 4 ANC (Table 4).

#### Predictors of women continuation to SBA after receiving optimal ANC (4 +) contacts

Factors predicting women continuation to the use of SBA after receiving optimal [4] ANC in pregnancy were determined from model 2 (ANC (4 +) and SBA) as shown in Tables 3 and 4. The result shows that all the factors (except partners’ education, getting money for medical help and urine sample taken in ANC) that were significant in model 1 were also significant in model 2 (including marital-status, distance to health facility and institutional delivery added to the model) (Tables 3 and 4). While women in maternal age 35–49 (UOR = 1.64, 95%CI = 1.51–1.77; AOR = 1.64, 95%CI = 1.29–1.95) are more likely to continue to the use of SBA after receiving 4 ANC, women residing in the rural (UOR = 0.19, 95%CI = 0.18–0.20; AOR = 0.75, 95%CI = 0.65–0.85) are less likely to continue to SBA after receiving 4 ANC (Tables 3 and 4).

Distance to health facility decrease the odds of continuation to SBA after receiving 4 ANC by almost half (48%) when other factors were unadjusted (UOR = 0.52, 95%CI = 0.48–0.55). The Odds of continuation to SBA otherwise increase when health decisions were made jointly (UOR = 4.38, 95%CI = 4.10–4.68; AOR = 1.26, 95%CI = 1.06–1.50) (Tables 3 and 4). Also, odds of continuation to SBA after 4 ANC increases and decreases by the rank of a healthcare provider when other factors were unadjusted and adjusted respectively. Similar to model 1, Women who received first ANC in second (UOR = 0.52, 95%CI = 0.44–0.62; AOR = 0.43, 95%CI = 0.40–0.47) and third (UOR = 0.08, 95%CI = 0.07–0.09; AOR = 0.08, 95%CI = 0.06–0.10) trimester are 2 and 13 times less likely to continue to SBA after receiving 4 ANC compared to those who received ANC in first trimester respectively (Tables 3 and 4). The odds of continuation to ANC increases when a woman took at least 2 doses of tetanus toxoid vaccine and had hospital delivery (Tables 3 and 4).

#### Predictors of women continuation to PNC after receiving optimal ANC (4 +) and using SBA at delivery (completion of the key continuum of care)

The predictive factors of PNC use after optimal ANC (4 +) contacts and SBA service were received were evaluated in model 3 (ANC (4 +), SBA and PNC) as presented in Tables 3 and 4. The result shows that other than marital status, all other predictors are significant in model 3 either under the unadjusted or adjusted (or both) association. Women from the southeast (UOR = 2.73, 95%CI = 2.26–3.32; AOR = 1.61, 95%CI = 1.29–2.01) and southwest (UOR = 2.73, 95%CI = 2.27–3.22; AOR = 1.68, 95%CI = 1.37–2.06) are approximately twice as likely as those from northcentral to continue to PNC after receiving 4 ANC and SBA (Tables 3 and 4). The odds of PNC use after receiving 4 ANC and SBA increases with women and their partners’ educational levels (Tables 3 and 4). Women in rich wealth quintiles increase the odds of PNC use after 4 ANC and SBA while women residing in the rural decrease the odds of PNC use after 4 ANC and SBA were received (Tables 3 and 4).

Taking at least 2 doses of tetanus toxoid vaccine (UOR = 9.17, 95%CI = 7.21–11.2) and checking blood pressures (UOR = 1.54,95%CI = 1.17–2.01) in ANC increase the odds of receiving PNC after 4 ANC and SBA by 817% and 54% respectively, but the odds decrease by 38% When getting money for medical help is a big problem (Tables 3 and 4). Institutional delivery increase and decrease the odds of receiving PNC after 4 ANC and SBA by 156% and 56% (UOR = 2.56, 95%CI = 2.29–2.85; AOR = 0.54, 95%CI = 0.47–0.62) respectively (Tables 3 and 4). Women who took iron folic acid in ANC (AOR = 1.98, 95%CI = 1.57–2.49) and had Caesarian delivery (AOR = 1.57, 95%CI = 1.28–1.91) are almost twice as likely as those who don’t to continue to PNC after receiving 4 ANC and SBA. Healthcare decisions made alone by women and ANC provided by doctors were strongly and positively associated with PNC use while the first ANC received in the second and third trimester were also strongly but negatively associated with PNC use respectively (Tables 3 and 4).

## Discussion

We investigated the gaps in the maternity continuum of care by evaluating the level of coverage and predictors of maternity continuation of care from pregnancy to childbirth and to the postpartum period in Nigeria. The goal is to inform a programming guide on designing improved MNCH intervention policy strategy, since CoC connects the essential maternal health services (ANC, SBA and PNC) and were assessed in this study based on the WHO recommendations for optimal care.

Coverage of antenatal care service in Nigeria has improved but has remained below the recommended 90% level as only 75% of pregnant women attended antenatal care at least once. It is however discouraging that barely 58% of pregnant women received at least four ANC contacts. This is in consonance with the report of the demographic health survey and a recent study on the sub-national analysis of optimal ANC utilization and satisfaction in Nigeria [10, 14, 43]. Continuation from optimal antenatal care to skilled delivery care service was observed in only over a third (37.4%) of the women while only one of every 15 (6.5%) pregnant women continued from ANC to SBA and PNC due to high dropout rate along the pathway of the continuum of care. Thus, there is more dropout between delivery and postnatal period than between pregnancy and childbirth period and therefore explain the irregular pattern in the continuation of care as reported in similar study in Nigeria and Ethiopia in SSA [6, 44].

Individual factors associated with women’s optimal ANC received in pregnancy are also associated with whether they utilized skilled delivery at birth and whether such women received postnatal care in the first 48 h after delivery. Hence the reason for the parallel identification of factors by the backward stepwise regression across the CoC model pathway. Women’s educational level, place of residence, region and wealth status were the socio-demographic and economic factors associated with the three essential maternal health service utilization while women’s healthcare decision-maker, provider of ANC, the timing of first ANC contact and number of tetanus toxoid vaccines taken as well as place (hospital) and mode (caesarian) of delivery were the associated interacting health system factors across the pathway of pregnancy to postpartum continuum and from delivery to post-delivery respectively. A similar factor has been identified by other studies in sub-Saharan Africa and Southeast Asia though with different statistical techniques (chi-square test of association) due to the comparability of women’s social-demographic, economic and health-related characteristics that includes quality of pregnancy care [6, 32, 33, 37].

Regardless of adjustment for women’s background characteristics, Women’s educational level is significantly associated with the use of ANC and continuation to the use of SBA and PNC. This implies that women with at least primary education are more likely to receive optimal ANC service and go on to use SBA and PNC at delivery and post-delivery respectively than those without any formal education. This aligns with studies that also found the significant effect of education on CoC in maternal health services [30, 33, 37]. Women residing in rural communities are however less likely to receive optimal ANC service, continue to use SBA at delivery and even complete the maternity continuum of care compared to those residing in the urban area. This can be attributed to a low level of education, preference for traditional births and the problem of accessibility and poor perception about primary healthcare service in the proximity of sub-Sahara African women [45]. Also, ANC provided by skilled and trained healthcare workers like; doctors, nurse/midwife was strongly and significantly associated with the maternity continuum of care across the 3 key maternity healthcare services (optimal ANC, SBA and PNC). Thus, women who received ANC from skilled providers have a higher likelihood of completing the care continuum than those who received care from unskilled providers. This highlighted the motivating impact of skilled healthcare providers on pregnancy outcomes compared to unskilled healthcare providers [46]. Also, in agreement with the study assessing women narrative of skilled delivery care provider and the implications for policy perspective in Nigeria and Ghana [47, 48].

Furthermore, geopolitical zone and socio-economic level positively influenced the use of optimal ANC, continuation to SBA and PNC as women from wealthier households and in the southern region (especially southeast and southwest) are more likely to receive and complete maternal health service than the poor women and those living in the northern region respectively. Inequality in the social-economic and geographical distribution of healthcare infrastructure has remained a militating factor of maternal health practice in Nigeria and Africa due to low coverage and selective health insurance package [21, 49, 50]. Late initiation of ANC (any time after the recommended first trimester) on the other hand negatively influence receiving optimal ANC, continuing to the use of SBA and PNC while receiving at least two tetanus vaccine during pregnancy increase the odds of receiving optimal ANC and sequentially completing the care of maternity continuum. The significance of ANC timing and tetanus vaccine status were also discovered in studies investigating the completion of maternity health services in Ethiopia [3, 5, 31]. We further observed that Women’s healthcare decision power is strongly associated with optimal ANC received, continuation to use of SBA and use of PNC. This is similar to findings from studies in Nigeria which reported the impact of women’s healthcare decision power on SBA use [13, 25] and comparable to a CoC study somewhere else that found an association between women’s healthcare decision power and the receipt of optimal ANC and continuation to use of PNC [37].

Maternal age and birth order (which decrease the odds of CoC as parity increases) were other significant predictors of optimal ANC receipt and continuation to use of SBA but not PNC. This finding was also reported in a study across 28 sub-Saharan African countries that investigated predictors of retentions in SBA after ANC service utilization [7]. Partners’ educational level and getting the money needed for medical help predicts women’s receipt of optimal ANC and PNC but not SBA. The problem of getting money for medical help is due to poor health insurance coverage and this along with the parity effect has been reported as determinant of ANC and optimal ANC use in a systematic review in SSA and a cross-sectional study in Nigeria respectively [10, 51].

Institutional delivery however predicts the use of SBA and subsequent use of PNC which was found to be significant in similar studies [33, 37, 52], Caesarian delivery is only associated with PNC visit. This can be recognized from the fact that hospital and caesarian delivery are assisted by skilled healthcare providers and most women who had caesarian birth will likely receive postnatal care to ensure recovery from the surgical site pain and to avoid infection as women with a normal vaginal delivery are more likely to have a better postnatal quality of life [53].

We further deduce that urine samples taken in ANC increase the chance of receiving optimal ANC while women who perceived distance to a health facility as a big problem are less likely to utilize SBA at delivery even if they receive optimal ANC. Which is in agreement with findings from similar studies in Nigeria and the Gambia [13, 32]. Blood pressure checked in ANC and iron-folic acid taken independently predict women’s continuation to use of PNC after receiving optimal ANC and SBA. However, marital status is not significantly associated with any of the CoC models which is in disagreement with the study that highlighted the significance of paternal influence on maternal health service utilization [54]. The model selection secluded factors assessing employment, health insurance, media exposure and blood sample taken in ANC across the maternity CoC, which was reportedly identified as predictors of SBA use [7, 25]. However, Differences in predictors of maternity CoC at different model stage across the pathway has been substantiated [33, 37].

## Conclusions

Despite encouraging turn-up at ANC, coverage of maternity continuum of care is low and below the WHO recommended level and standards in Nigeria. Less than three-fifths, two-fifths and one-fifteenth of pregnant women received optimal ANC, continue to the use of SBA and PNC respectively. The dropout rate across the continuum of care model pathway is alarming and needs an urgent revisit. There is however more dropout at the PNC than SBA and at the optimal ANC. Educational attainment, place of residence, geopolitical zone and wealth status were the joint socio-demographic predictors while women’s healthcare decision power, skilled ANC provider, the timing of first ANC and number of tetanus toxoid vaccines taken in ANC were the equivalent health-related predictors of the key maternity continuum of care. While maternal age and parity were associated with the continuation of care from ANC to SBA, partners’ education and medical finances were associated with PNC continuity after ANC. Hospital delivery predicts the use of SBA and continuation to PNC while Caesarian delivery influences the use of PNC.

### Study strengths and limitations

It is not improbable that the study suffers from responder bias since the data quality depends on respondents’ ability to recall events in the last five years preceding the survey. The study investigated the association between women’s characteristics and maternity CoC and does not infer that these factors are causes of maternity CoC due to the cross-sectional design of the data. Therefore, interpretation should be limited to the association. The application of secondary data posed the difficulty of data incompleteness and restricted the authors to the choice of the available set of independent factors assessed in the survey, which was minimized by the analysis of a weighted sample of women with at least one birth in the last five years preceding the survey and the automated model selection approach. Non-availability of variables or data to assess the type of healthcare facility or women first place of care which experience could be the reason for dropout or incomplete CoC was also a limitation. However, the study strength can be observed from the application of a nationally representative sample which increases the study generalizability. The fact that we adjust for complex survey design based on the sample weighting, clustering and stratification improves the reliability of the study findings and accuracy therein. Furthermore, this is the first study that assessed the maternity CoC completion in Nigeria using the well-known and utilized WHO standards on a minimum of 4 ANC, SBA by doctor/nurse/midwife and first PNC within the first 48 h and therefore presents an opportunity to strategize towards transitioning into the newly recommended minimum of 8 ANC contacts and achieving the SDG-3 in Nigeria.

### Recommendations

We infer from the study findings that, coverage of the three essentials maternity continuum of care (ANC, SBA and PNC) in Nigeria is below the 90% recommendation, which will halt the attainment of the 2030 SDG on improving childhood health in Nigeria. A centralized strategy that will improve MNCH program practice is however required to unify national programs and breach the coverage gap. Governmental and nongovernmental agencies need to be steadfast in providing improved support for sensitization programs around early ANC initiation and an optimal number of ANC visits (the minimum of 4 and transition into 8). Improved ANC packages that strengthen women in; pregnancy care, healthcare decision power and educational awareness for childbirth preparedness are recommended to improve pregnancy outcomes. Capacity building for pregnant women to improve the use of SBA at delivery and the least utilized PNC at post-delivery is also vital to optimize mother and child survival. Contextual research investigating the reason for dropout and non-compliance with the WHO recommendations of the maternity continuum of care is required to better provide intervention strategy to improve on the low completion coverage.

## Data Availability

The anonymized data is available in the public domain. Dataset used (generated and/or analyzed) in this current study are available on reasonable request from the corresponding author, at www.dhsprogram.com and in the DHS program open repository http://dhsprogram.com/pubs/pdf/FR359/FR359.pdf.

## Abbreviations

ANC: Antenatal Care
CoC: Continuum of Care
CI: Confidence Interval
FCT: Federal Capital Territory
LMICs: Lower-Middle-Income Countries
MMR: Maternal Mortality Ratio
MNCH: Maternal Newborn and Child Health
NPHC: National Population and Housing Census
NMR: Neonatal Mortality Rate
NDHS: Nigerian Demographic and Health Survey
PNC: Postnatal Care
PRMR: Pregnancy-related Mortality Ratio
SBA: Skilled Birth Attendants
SSA: Sub-Saharan Africa
SDG: Sustainable Development Goals
WHO: World Health Organization
